# Alpha peak activity in resting-state EEG is associated with depressive score

**DOI:** 10.3389/fnins.2023.1057908

**Published:** 2023-03-07

**Authors:** Peng Zhou, Qian Wu, Liying Zhan, Zhihan Guo, Chaolun Wang, Shanze Wang, Qing Yang, Jiating Lin, Fangyuan Zhang, Lu Liu, Dehui Lin, Wenbin Fu, Xiang Wu

**Affiliations:** ^1^Bao’an Traditional Chinese Medicine Hospital, Shenzhen, China; ^2^Sanming Project of Medicine in Shenzhen, Fuwenbin’s Acupuncture and Moxibustion Team of Guangdong Provincial Hospital of Chinese Medicine, Shenzhen, China; ^3^Department of Acupuncture and Moxibustion, Guangdong Provincial Hospital of Chinese Medicine, The Second Affiliated Hospital of Guangzhou University of Chinese Medicine, Guangzhou, China; ^4^Department of Psychology, Sun Yat-sen University, Guangzhou, China; ^5^Second Clinical College, Guangzhou University of Chinese Medicine, Guangzhou, China; ^6^Clinical Medical College of Acupuncture Moxibustion and Rehabilitation, Guangzhou University of Chinese Medicine, Guangzhou, China; ^7^Innovative Research Team of Acupuncture for Depression and Related Disorders, Guangdong Provincial Hospital of Chinese Medicine, The Second Affiliated Hospital of Guangzhou University of Chinese Medicine, Guangzhou, China

**Keywords:** alpha peak activity, depression, frequency spectrum, resting-state EEG, correlation analysis

## Abstract

**Introduction:**

Depression is a serious psychiatric disorder characterized by prolonged sadness, loss of interest or pleasure. The dominant alpha peak activity in resting-state EEG is suggested to be an intrinsic neural marker for diagnosis of mental disorders.

**Methods:**

To investigate an association between alpha peak activity and depression severity, the present study recorded resting-state EEG (EGI 128 channels, off-line average reference, source reconstruction by a distributed inverse method with the sLORETA normalization, parcellation of 68 Desikan–Killiany regions) from 155 patients with depression (42 males, mean age 35 years) and acquired patients’ scores of Self-Rating Depression Scales. We measured both the alpha peak amplitude that is more related to synchronous neural discharging and the alpha peak frequency that is more associated with brain metabolism.

**Results:**

The results showed that over widely distributed brain regions, individual patients’ alpha peak amplitudes were negatively correlated with their depressive scores, and individual patients’ alpha peak frequencies were positively correlated with their depressive scores.

**Discussion:**

These results reveal that alpha peak amplitude and frequency are associated with self-rating depressive score in different manners, and the finding suggests the potential of alpha peak activity in resting-state EEG acting as an important neural factor in evaluation of depression severity in supplement to diagnosis.

## 1. Introduction

Depression is characterized by prolonged sadness, loss of interest, difficulty to experience pleasure, and possibly leads to suicide in extreme cases (Friedrich, 2017). Globally, over 300 million people are suffering from this serious neurological disorder (World Health Organization, 2017). In clinical practice, the diagnosis of depression faces difficulties. The first is the complex symptoms of depression, particularly the combined symptoms with other disorders such as addictive disorders and anxiety disorders (for reviews, see Tiller, 2013; Emery and Akil, 2020). A categorical distinction between depression and other psychological disorders remains ambiguous and antidepressant drugs are sometimes effective for unrelated disorders (Kalat, 2015). In this regard, measurement scales such as the Self-Rating Depression Scales (SDS) are valuable to help clinicians further evaluate symptoms and better identify, diagnose, and treat depression in practice (Gelenberg, 2010). Second, the depression diagnosis lacks quantitative measures that are required to further evaluate the severity of patients, and the SDS is such a test that provides an evaluation of the severity of depressive symptom. Regarding this, in supplement to a diagnosis of patients vs. healthy controls, the SDS measure allows further quantitative assessment of the severity of depressive symptoms of patients.

The human electroencephalogram (EEG) measures oscillating brain activity in a broad frequency range typically including the delta (1–3 Hz), theta (4–6 Hz), alpha (7–13 Hz), beta (14–30 Hz), and gamma (30–100 Hz) bands, and alpha activity is the most prominent rhythmic activity and is manifested by a peak (∼10 Hz) in the frequency spectrum (Klimesch, 1999). Deferent measurements have been adopted to quantify the characteristics of activities in the alpha range (Klimesch, 1999; Pfurtscheller and da Silva, 1999; Angelakis et al., 2004). In terms of dividing the alpha spectrum into sub-bands, low (8–9 Hz) and high (11–12 Hz) alpha bands that are below and above the peak frequency of alpha activity have been suggested and are assumed to be generated by different pacemakers with different cortical/thalamic origins. Besides the best-known alpha rhythm that is widely distributed with larger amplitudes over posterior regions, the rolandic mu (mu stands for motor) rhythm manifests over the motor cortex and the auditory tau (tau stands for temporal) rhythm is best seen over the auditory cortex. The alpha activity investigated in the present study referred to the classic alpha activity. An alternative way to observe alpha activity is regarding individual alpha peaks, i.e., focusing on analyzing the characteristics of alpha peak activity. After identifying individual alpha peaks, two measures of alpha peak activity are typically investigated. Alpha peak amplitude assesses amplitude of activity around alpha peak. While alpha band amplitude and alpha peak amplitude both measure amplitude of alpha activity, the former is applied for a defined alpha frequency range and the latter emphasizes spectrum characteristics around individual alpha peaks. Alpha peak amplitude is suggested to reflect synchronous neural discharging and enhanced alpha peak amplitude is considered as an indicator of cortical hypoactivity (Klimesch, 1999). Another major measure of alpha peak activity is alpha peak frequency, which is the frequency at which the strongest spectrum activity appears. It has been observed that alpha peak frequency is associated with cerebral blood flow and oxygenation. For example, alpha peak frequency positively correlated with cerebral blood flow (Jann et al., 2010) and decreased after hypoxia with hemoglobin oxygen (Van der Worp et al., 1991). Alpha peak frequency is assumed to be related to brain metabolism and is considered as an indicator of cognitive preparedness that reflects the brain’s capacity for optimal cognitive performance (Angelakis et al., 2004). The amplitude and frequency of alpha peak activity vary across individuals and have been shown to be associated with a broad range of cognitive functions and states (Angelakis et al., 2004; Cecere et al., 2015; Ronconi and Melcher, 2017). Individual alpha peak amplitudes and frequencies of the same subjects were also found to be reliable across examination sessions on separate days using intraclass correlation coefficient (Katyal et al., 2019). The dominant alpha peak activity in resting-state EEG has been suggested to be a potential intrinsic neuromarker of diagnosis of mental disorders (Klimesch, 1999; Lefebvre et al., 2018; Newson and Thiagarajan, 2019).

A large body of depression research has investigated EEG band amplitudes in different frequency ranges. The results generally found elevated amplitude of patients compared to normal controls in the theta, alpha, and gamma bands, though some did not observe the increased amplitude or found decreased amplitude in patients (for reviews, see de Aguiar Neto and Rosa, 2019; Fernández-Palleiro et al., 2019; Schiller, 2019). Among the frequency ranges, alpha peak activity is of particular interest, partly due to the early proposal of its relation with affective processing “The ability to produce “good” alpha waves seems to be a neurophysiological characteristic which is related in some way to the affective capacity of the individual” (Lemere, 1936). Increased amplitude in the alpha frequency range (∼7–13 Hz) has been generally found in depression patients compared to healthy controls (for review, see Olbrich and Arns, 2013). Moreover, the alpha amplitude of patients vs. healthy controls has been found to exhibit hemispheric asymmetries, generally with relatively stronger alpha amplitude over left then right frontal regions, as well as relatively stronger right parietotemporal alpha amplitude (for reviews, see Bruder et al., 2017; Van der Vinne et al., 2017; Kaiser et al., 2018). Despite the extensive investigation of comparing alpha band amplitudes between patients and normal controls, the research that further evaluates severity of depression patients by investigating the relationship between individual alpha peak activities and depression severities is sparse and existing understanding is largely ambiguous. A related clue was from a recent non-clinical study that used resting-state EEG with 19 electrodes and investigated correlations between alpha peak activities and self-report depressive scores of non-clinical subjects (Tement et al., 2016). The results did not show a significant correlation between alpha peak amplitudes and depressive scores, although for females there was a non-significant trend of a negative correlation. For alpha peak frequency, a significant correlation was also lacking for the overall subjects, despite that the results suggested a modulation by gender that there was a significant positive correlation for males and a significant negative correlation for females.

Therefore, the present study aimed to examine the association between individual depressive patients’ alpha peak activities and the severities of their depressive symptoms. We recruited 155 depressive patients and recorded their 5-min resting-state EEG. Correlation analysis was performed between the alpha peak measures including alpha peak amplitude and alpha peak frequency and the patients’ SDS scores. As introduced above, assessment of the SDS score allowed further evaluation of individual patients’ depression severities. Accordingly, analysis of alpha peak activity focused on individual characteristics of alpha activity, and the measures of alpha peak amplitude and alpha peak frequency provided examination in terms of different physiological mechanisms. Moreover, the EEG source reconstruction research has recently made considerable progresses in localizing cortical sources of resting-state scalp EEG signals and overcoming the issue of correlated signals from scalp electrodes (Schoffelen and Gross, 2009; Niso et al., 2019), which would be of particular importance when the accuracy and efficiency of a neuromarker are under concern in clinical applications. The EEG data in the present study were thus analyzed on the cortical source level.

Together, given the role of alpha peak activity in diagnosing mental disorders, we hypothesize that alpha peak activity is an important factor for evaluation of depression severity in supplement to diagnosis and predict that individual patients’ alpha peak measures in resting-state EEG would correlate with their depressive scores.

## 2. Materials and methods

### 2.1. Participants

Totally 155 patients with depression (42 males; mean age 35.14 years, *SD* = 11.98) were recruited from Guangdong Provincial Hospital of Chinese Medicine, China. The patients were diagnosed depressive episodes according to the International Classification of Diseases 10th Edition (ICD-10, F32) by professional psychiatrists. [Note that major depressive disorder is the diagnosis according to the Diagnostic and Statistical Manual of Mental Disorders 5th Edition (DSM-5)]. The patients were recruited with the following criteria. Inclusion criteria: (1) Aged between 18 and 65 years; (2) Diagnosed with a first appearance depressive disorder; (3) Have not received medications for depression within 12 weeks prior to enrollment in the study. Exclusion criteria: (1) Have a tendency to commit suicide (be evaluated by psychiatric specialists); (2) Have participated in other clinical trials within 4 weeks before the start of the study; (3) A history of severe cardiac, pulmonary, hepatic, and renal disease requiring treatment; (4) The patient was a pregnant or lactating woman; (5) Who install a pacemaker or an artificial joint; (6) Addiction to drugs or alcohol; (7) Taking antidepressants or the pharmacological effects of such anti-depressants had not been washed out. The SDS scores (Zung, 1965) were obtained for 154 among the 155 patients.

The research protocols in this study followed the tenets of the Declaration of Helsinki and were approved by the Ethics Committee of Guangdong Provincial Hospital of Chinese Medicine (YF-2020-198-01) and the Institutional Review Board of Psychology Department of Sun Yat-sen University. All participants provided written informed consent before their participation.

### 2.2. Data acquisition and analysis

The participants were asked to sit and relax for 5 min with their eyes closed during the EEG recording. An EGI (Electrical Geodesic Inc., OR, USA) system with a 128 electrode Hydro Cel Geodesic Sensor Net was used to record the EEG. The EEG was digitized at 1,000 Hz and filtered with a 0.05–100 Hz band-pass filter. The electrodes were referenced to CZ. Electrodes impedances were kept below 50 kΩ.

A prior power analysis was performed using G*Power 3 (Faul et al., 2007). The analysis was in accordance to the effects reported in a previous study (Tement et al., 2016). The mean effect size for electrodes showing significant correlation between alpha peak frequency and depressive score was 0.32 for males and −0.31 for females. Based on the smaller effect size of −0.31, the alpha level of *p* < 0.05 (two tailed), and the power of 0.80, 79 subjects were required.

The EEG data were analyzed by a customized processing routine involving the usage of MATLAB (The Mathworks, Natick, MA, USA), EEGLAB (for artifact removal with Independent Component Analysis),^1^ Brainstorm (for source reconstruction),^2^ and mfeeg (for basic EEG signal processing).^3^ The EEG was down-sampled to 100 Hz and 0.5–45 Hz band-pass filtered. Bad electrodes were determined by a Clean Rawdata procedure in EEGLAB (rejecting flat channels, channels with a large amount of noise based on their standard deviation, and channels poorly correlated with other channels) and then interpolated with data from neighboring electrodes. The Infomax Independent Components Analysis (ICA) module in EEGLAB was used to decompose the EEG and artifact components (including ocular and muscle artifact) were removed (Delorme et al., 2007; Pion-Tonachini et al., 2019). Data quality was further examined *via* a manual checking procedure, in which data from one patient were excluded due to large signal noise. Finally, EEG and clinical data of 153 among the 155 recruited patients were subjected into the following analyses. The EEG was then re-referenced to the common average reference. Source reconstruction was conducted using the forward and inverse models implemented in Brainstorm (Tadel et al., 2011). The strength of the source transformed signal was under the sLORETA normalized unit. The cortical surface was parcellated into 68 brain regions with the Desikan-Killiany atlas (Desikan et al., 2006), and source time series of the brain regions were extracted by averaging vertex time series in each region. The frequency spectra of the time series (68 time series for 68 source regions) were computed by the Fast Fourier transform (FFT). The amplitude was transformed to a log_10_ scale and the spectrum was smoothed with a 0.17 Hz moving-average window. For each participant, we obtained the alpha peak frequency with maximum amplitude between 7 and 13 Hz *via* a customized automatic procedure utilizing MATLAB function findpeaks, and calculated the alpha peak amplitude as the average amplitude within ±1 Hz around the alpha peak frequency (Klimesch, 1999; Tement et al., 2016). BrainNet Viewer was used to present the locations of source regions on a brain template (Xia et al., 2013).

An evaluation procedure was conducted to identify the source regions that showed valid alpha peak activity. For each of the source regions, a *t*-test was performed on alpha peak amplitude relative to the average amplitude of one neighboring frequencies on either side (Norcia et al., 2015), and a valid alpha peak was indicated by alpha peak amplitude being significantly larger than the amplitudes of surrounding frequencies (*p* < 0.01).

The correlation analyses were conducted using the parametric Pearson or non-parametric Spearman correlation method depending on the normality of data. Confounding effects of age and gender were controlled for by partial correlation (the present study focused on a direct correlation between EEG and depressive measures, see the Supplementary material for further analyses of effects of gender and age. In brief, for gender, the results showed a negative correlation between alpha peak amplitude and depressive score in females; for age, a negative correlation was observed between alpha peak amplitude and age and between alpha peak frequency and age). False discovery rate corrections (Storey, 2002) were applied to the results and corrected *p*-values < 0.05 were considered significant.

## 3. Results

### 3.1. Self-rating depression scales score

**FIGURE 1:**
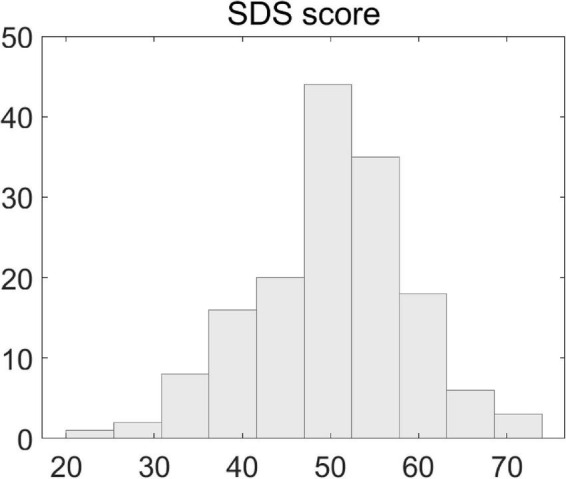
Illustration of scores of Self-Rating Depression Scales. Shown is the histogram distribution of individual patients’ SDS scores.

The SDS scores of the patients ranged from 22 to 74. The histogram distribution of individual scores is illustrated in Figure 1 and the Kolmogorov–Smimov (K-S) test indicated that the data satisfied the normality assumption (K-S *p* > 0.05).

### 3.2. Alpha peak activity

**FIGURE 2:**
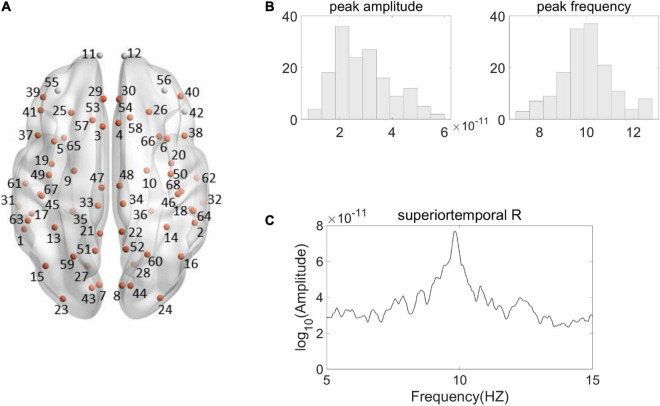
Illustration of Alpha peak activity in the frequency spectrum. **(A)** Shown are the 68 Desikan–Killiany regions from a top view, among which the source regions showing valid alpha peak activity are indicated by the orange color. (The name and the number of the regions are according to the standard Desikan–Killiany atlas, see Supplementary Table 1 for further details). **(B)** Shown are the histogram distributions of individual alpha peak amplitudes (left) and frequencies (right) from a representative region (right superior temporal). **(C)** The spectrum of the representative region from a representative participant is shown. The source signal strength was under the sLORETA normalized unit.

For alpha peak activity, we first carried out an evaluation procedure to identify the source regions that showed significantly larger amplitude of alpha peak activity relative to the amplitudes of surrounding frequencies. Valid alpha peak activity was found in 63 source regions (Figure 2A). Only the source regions that showed valid alpha peaks entered into the following correlation analyses.

### 3.3. Correlation between EEG and clinical measures

We then performed correlation analyses between the EEG measures and clinical scores. Because the data of alpha peak amplitude were not always normally distributed [the amplitude data satisfied the normality assumption (K-S *p* > 0.05) in 48 among the 68 regions], we conducted the correlation analyses using the Spearman correlation method. The Pearson correlation method was used for alpha peak frequency [the frequency data satisfied the normality assumption (K-S *p* > 0.05) in all the 68 regions].

**FIGURE 3:**
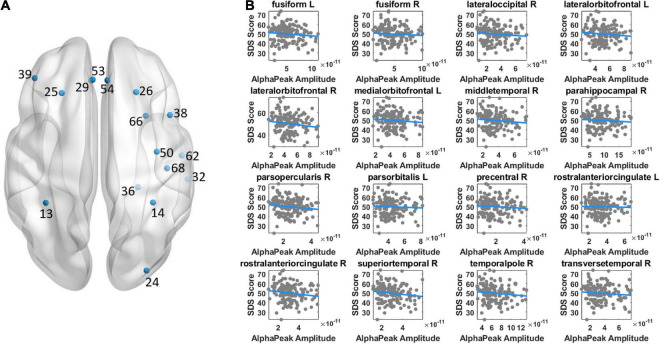
Illustration of correlation results between alpha peak amplitude and depressive score. **(A)** The source regions that showed significant correlations are indicated by colored points. Positive or negative correlations are indicated by the red or blue color, respectively. **(B)** Shown are the scatter plots of significant correlations at individual regions. The color of the fitted line indicates positive (red) or negative (blue) correlation. The results revealed a negative correlation between alpha peak amplitude and depressive score. Other conventions are as in Figure 2.

Both alpha peak amplitude and alpha peak frequency were found to be significantly correlated with the SDS score, and the results further revealed different correlation patterns for alpha peak amplitude and frequency. For alpha peak amplitude, significant negative correlations were found at 16 source regions (*p*_corrected_ < 0.05), including the bilateral fusiform, right lateral occipital, bilateral lateral orbitofrontal, left medial orbitofrontal, right middle temporal, right parahippocampal, right pars opercularis, left pars orbitalis, right precentral, bilateral rostral anterior cingulate, right temporal pole, right superior temporal and right transverse temporal areas (Figure 3 and Table 1). Moreover, for each region that showed significant correlation on either hemisphere, Table 2 lists a right/left asymmetry ratio that represent the ratio between correlation *r*-values of the right and left hemispheres (Bouchard et al., 1990).

**TABLE 1:** Significant correlation *r*-values between alpha peak amplitude and depressive score.

Region name	*r*-value	Region name	*r*-value
Fusiform L	−0.19	Pars opercularis R	−0.20
Fusiform R	−0.23	Pars orbitalis L	−0.18
Lateral occipital R	−0.20	Precentral R	−0.21
Lateral orbitofrontal L	−0.21	Rostral anterior cingulate L	−0.20
Lateral orbitofrontal R	−0.24	Rostral anterior cingulate R	−0.28
Medial orbitofrontal L	−0.26	Superior temporal R	−0.27
Middle temporal R	−0.27	Temporal pole R	−0.25
Parahippocampal R	−0.21	Transverse temporal R	−0.20

**TABLE 2:** *R*-values of the left and right hemispheres and the right/left asymmetry ratio for each region showing significant correlation between alpha peak amplitude and depressive score on either hemisphere.

Region name	Left	Right	Right/left ratio
Fusiform	−0.19	−0.23	1.21
Lateral occipital	−0.04	−0.20	5.00
Lateral orbitofrontal	−0.21	−0.24	1.14
Medial orbitofrontal	−0.26	−0.15	0.58
Middle temporal	−0.09	−0.27	3.00
Parahippocampal	−0.14	−0.21	1.50
Pars opercularis	−0.11	−0.20	1.82
Pars orbitalis	−0.18	−0.13	0.72
Precentral	0.00	−0.21	397.03
Rostral anterior cingulate	−0.20	−0.28	1.40
Superior temporal	−0.06	−0.27	4.50
Temporal pole	−0.11	−0.25	2.27
Transverse temporal	−0.06	−0.20	3.33

A right/left ratio larger than one indicates greater correlation on the right hemisphere and a right/left ratio smaller than one indicates greater correlation on the left hemisphere. (That for the precentral region, the extremely large right/left ratio was due to the fact that the r-value of the left hemisphere was nearly zero).

**FIGURE 4:**
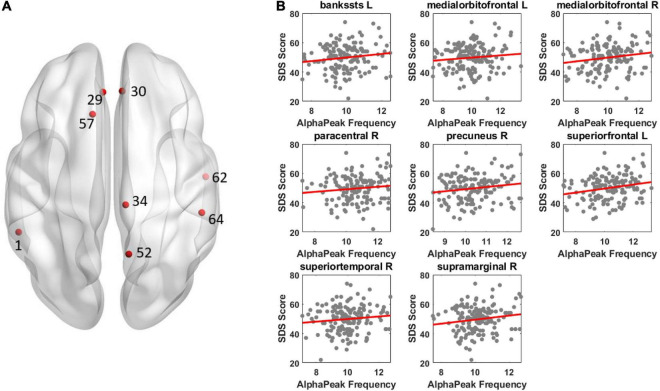
Illustration of correlation results between alpha peak frequency and depressive score. The results revealed a positive correlation between alpha peak frequency and depressive score. Other conventions are as in Figure 3.

For alpha peak frequency, the results showed significant positive correlations at 8 source regions (*p*_corrected_ < 0.05) including the left bankssts, bilateral medial orbitofrontal, right paracentral, right precuneus, left superior frontal, right superior temporal and right supramarginal areas (Figure 4 and Table 3). Table 4 further lists the right/left asymmetry ratio for each region that showed significant correlation on either hemisphere.

**TABLE 3:** Significant correlation *r*-values between alpha peak frequency and depressive score.

Region name	*r*-value	Region name	*r*-value
Van kssts L	0.25	Precuneus R	0.19
Medial orbitofrontal L	0.18	Superior frontal L	0.29
Medial orbitofrontal R	0.23	Superior temporal R	0.21
Paracentral R	0.19	Supramarginal R	0.19

**TABLE 4:** *R*-values of the left and right hemispheres and the right/left asymmetry ratio for each region showing significant correlation between alpha peak frequency and depressive score on either hemisphere.

Region name	Left	Right	Right/left ratio
Ban kssts	0.25	0.11	0.44
Medial orbitofrontal	0.18	0.23	1.28
Paracentral	0.12	0.19	1.58
Precuneus	0.04	0.19	4.75
Superior frontal	0.29	0.17	0.59
Superior temporal	0.16	0.21	1.31
Supramarginal	0.13	0.19	1.46

Conventions are as in Table 2.

## 4. Discussion

The present study examined the association between alpha peak activity of resting-state EEG and depressive scores of patients with depression. Over widely distributed brain regions, a significant negative correlation was found between individual patients’ alpha peak amplitudes and SDS scores and a significant positive correlation was found between individual patients’ alpha peak frequencies and SDS scores.

Alpha peak amplitude and frequency have been commonly reported to correlate with a behavioral measure in distinct manners and are considered to have different psychophysiological significances (Klimesch, 1999; Tement et al., 2016; Katyal et al., 2019). For example, in a visual perceptual task, alpha peak frequency but not alpha peak amplitude showed a significant correlation with perceptual performance (Katyal et al., 2019). For depression related research, as described in the Introduction, Tement et al. (2016) found that alpha peak amplitude and frequency correlated with depressive scores in different manners although the effects were largely related to a gender modulation. It appeared that a negative correlation between alpha peak amplitude and depressive score was observed in both the results of Tement et al. (2016) and the present results. Note that the two studies have differences in many aspects. For instance, Tement et al. (2016) involved non-clinical students and the present study investigated depressive patients. The depression scales in the study of Tement et al. (2016) is the Center for Epidemiologic Studies Depression Scale (CES–D) (Radloff, 1977). Both of the CES-D and the SDS were commonly used depression scales. While the CES–D depicts symptoms that are associated with depression, the measure of the SDS reflects common symptoms of depression (Umegaki and Todo, 2017) and was preferred in our clinical practice. It would be also emphasized that the present study focused on a direct association between alpha peak activity and depressive score, with the possible effects of age and gender being controlled for. Therefore, while the two studies are supplementary and indicative to each other in improving our understanding of the relationship between alpha peak activity and depression, direct comparison between the two studies might be inappropriate.

For the psychophysiological backgrounds and significances of alpha peak amplitude and frequency, alpha peak amplitude is suggested to be more associated with synchronous neural discharging (Klimesch, 1999) and alpha peak frequency is considered to be more related to brain metabolism (Klimesch, 1999; Angelakis et al., 2004). Angelakis et al. (2004) suggests that alpha peak frequency should not be compared to alpha peak amplitude. In other words, for the prominent alpha peak activity in the human EEG, alpha peak amplitude and frequency provide measures based on different physiological mechanisms. Therefore, the observed differences in the correlation patterns between alpha peak amplitude and frequency reflect different physiological substrates (which should not be interpreted with regard to incompatible/compatible results). Specifically, for the clinical application of identifying a potential neuromarker for the severity of depression patients, alpha peak amplitude and frequency would provide valuable contributions in terms of different physiological mechanisms. One concern could be whether the different correlation patterns between alpha peak amplitude and frequency may result from a negative relation between the amplitude and frequency themselves. There was a significant negative correlation between alpha peak amplitude and frequency (see Supplementary Table 5). However, it is worth noting that in statistics, correlations between data A and C and between data B and C do not necessarily result from a correlation between data A and B. Accordingly in the current empirical data, the 16 regions showing significant negative correlation for alpha peak amplitude and the eight regions showing significant positive correlation for alpha peak frequency only overlapped in two regions. In other words, if a significant negative correlation between amplitude and frequency at a region could result in a significant negative correlation between the amplitude and clinical score and a significant positive correlation between the frequency and clinical score at that region, it explained data of only two regions. Therefore, the correlation between alpha peak amplitude and frequency was unlikely a major factor for the present observation. As discussed above, instead of direct comparison between the two measures, alpha amplitude and frequency are suggested to be investigated with respect to their different physiological significances (Angelakis et al., 2004).

We adopted the source reconstruction approach with a particular interest to identify cortical locus of alpha peak activity that would be associated with depression severity. This could have significant implications for clinical applications, such as when more precise cortical localization is under consideration in diagnosis of depression. The present results indicated the involvement of a wide brain network including the frontal, parietal, temporal, as well as occipital regions in depression, which is consistent with the reported engagement of widely distributed brain areas in depression research using resting-state functional Magnetic Resonance Imaging (fMRI) approach (Wu et al., 2011; Pizzagalli, 2014; Gong and He, 2015; Cheng et al., 2018; Malhi and Mann, 2018; Shi et al., 2020; Wang et al., 2020). Despite different physiological sources of fMRI and EEG signals (indirect measure of neuronal activity for fMRI; direct measure of electrophysiological activity for EEG which allows investigation of frequency-specific oscillatory activity), analysis and comparison of cortical regions and networks of fMRI and EEG activities (for EEG, indicated by source reconstruction) would be indicative (Hipp et al., 2012). Future research of alpha peak activity is recommended to adopt the source reconstruction approach, particularly when clinical applications are involved. Moreover, previous studies comparing alpha band amplitude between patients and normal controls have shown relatively stronger left frontal and right parietotemporal alpha amplitude in patients (see Bruder et al., 2017; Van der Vinne et al., 2017; Kaiser et al., 2018). The present patients’ correlation results between the alpha peak amplitude and SDS score appeared to show similar asymmetry patterns, through mainly for parietotemporal areas (Figure 3 and Table 2): the right/left ratio was smaller than 1 at 2 left frontal regions and was larger than 1 at 6 right parietotemporal regions; whereas was larger than 1 at 3 right frontal regions and was smaller than 1 at 0 left parietotemporal region. In addition, similar patterns were also observed for the correlation of alpha peak frequency (Figure 4 and Table 4), the right/left ratio was smaller than 1 at 1 left frontal region and was larger than 1 at 3 right parietotemporal regions; whereas was larger than 1 at 2 right frontal regions and was smaller than 1 at 1 left parietotemporal region.

Either the eye-closed condition or the eye-open condition has been adopted in resting-state EEG depression studies investigating alpha activity (Van der Vinne et al., 2017; Newson and Thiagarajan, 2019). EEG alpha activity is highest in the eyes-closed condition and is reduced when eyes open or engaged in attention-demanding tasks. Relative to the resting-state alpha activity with eyes closed that indicates a baseline state of the brain unbiased by any task, the reduction of alpha activity reflects higher levels of attention or arousal states (Klimesch, 1999; Fingelkurts and Fingelkurts, 2015; Katyal et al., 2019). In previous resting-state EEG depression studies, the increased alpha amplitude in depression patients compared to healthy controls and the alpha asymmetry have been observed in both the eye-closed and eye-open conditions (Van der Vinne et al., 2017). A resting-state EEG study investigating correlations between alpha peak activities and depressive scores of non-clinical subjects also used the eye-closed condition (Tement et al., 2016). Moreover, considering the long-time 5 min EEG recoding of our clinical patients, the more relaxed eye-closed condition would help avoid extra burden and fatigue and prevent potential large EEG signal noise (Fingelkurts and Fingelkurts, 2015; Barry and De Blasio, 2017). Therefore, the eye-closed condition was adopted in the current study.

The present study has the following limitations. The first is the lack of a group of healthy control subjects that matched in gender and sex and had a comparable sample size, and the current study did not carry out a replication analysis for the prior finding of greater alpha amplitude for patients vs. normal controls. Second, the present study conducted diagnosis of depression. Disorders that have combined symptoms with depression, such as anxiety disorders, were not separately diagnosed. The present results would be to some extent associated with the symptoms of disorders related to depression. Accordingly, it is worth noting that the previous finding of greater alpha amplitude for patients vs. healthy controls and the present result of the negative relationship between alpha amplitude and the SDS score were supplementary, rather than conflicting, to each other. As have been discussed, the depression diagnosis involves considerations of combined symptoms and the SDS score is a further evaluation of depressive symptoms supplementary to the diagnosis. Particularly, it has been hypothesized that anxious arousal in patients is associated with right parietotemporal hyperactivation and studies have found that depression patients having a comorbid anxiety disorder showed less alpha amplitude over the right than left parietal sites (for review, see Bruder et al., 2017). Future research is required to further explore the relationship between depression and related disorders, and studies should relate alpha activity to not only depression but also severity of anxiety or other symptoms. Meantime, further attempts are also required to adopt or formulate distinct behavior and neural measures, for an improved understanding of the neural substrates of patients’ depressive symptoms.

## Conclusion

In conclusion, the present results showed an association between alpha peak activity in resting-state EEG and the severity of depressive symptom. These findings indicate a potential role of alpha peak activity in evaluation of depression severity in supplement to diagnosis.

## Data availability statement

The data generated during and/or analyzed during the current study are available from the corresponding author on reasonable request.

## Ethics statement

The studies involving human participants were reviewed and approved by the Ethics Committee of Guangdong Provincial Hospital of Chinese Medicine (YF-2020-198-01) and the Institutional Review Board of Psychology Department of Sun Yat-sen University. The patients/participants provided their written informed consent to participate in this study.

## Author contributions

XW conceived the research. QW, LZ, WF, and XW designed the research. QW, ZG, LZ, CW, DL, SW, QY, JL, FZ, LL, and PZ collected the data. LZ, CW, QW, ZG, DL, SW, QY, JL, FZ, and LL analyzed the data. PZ was involved in the discussion of the data. PZ, WF, and XW were responsible for project supervision and funding acquisition. QW, LZ, CW, and XW wrote the manuscript. All authors commented on and edited the manuscript and approved the final version of the manuscript.

## Footnotes

1
https://sccn.ucsd.edu/eeglab

2
https://neuroimage.usc.edu/brainstorm

3
http://sourceforge.net/p/mfeeg
