# Supplementary material for: Alpha peak activity in resting-state EEG is associated with depressive score
Source: Front Neurosci. 2023 Mar 7;17:1057908. doi: 10.3389/fnins.2023.1057908 (PMC10027937; doi:10.3389/fnins.2023.1057908)
Supplement: Supplementary file 1 [file Data_Sheet_1.docx]

**Supplementary Materials**

Fig. S1-S3; Table S1-S5; Supplementary Text


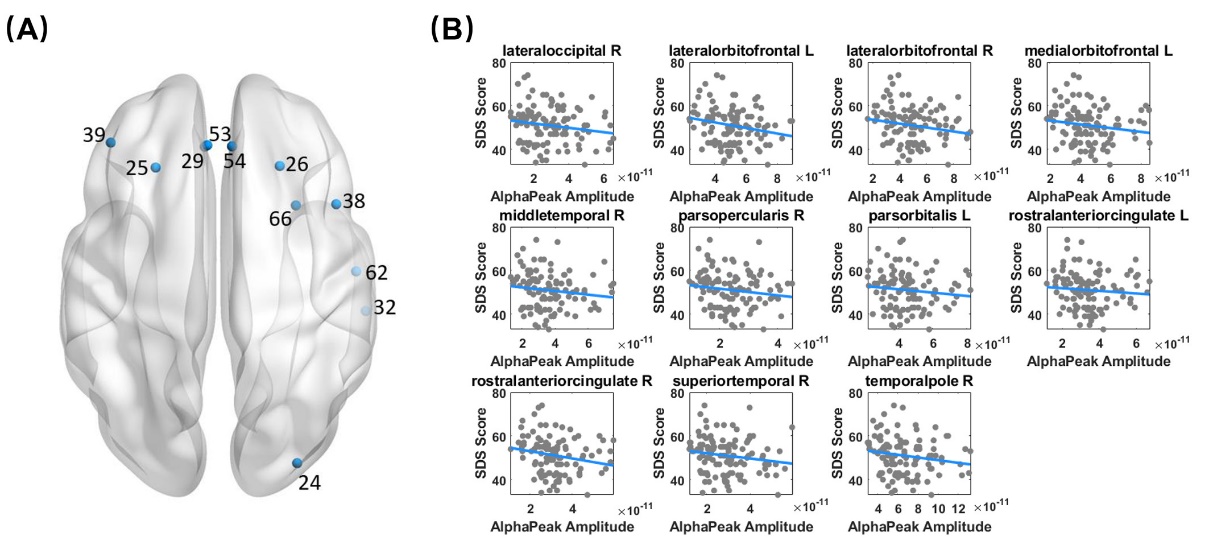


**Fig. S1**. **Illustration of correlation results between alpha peak amplitude and depressive score in females**. The results revealed a negative correlation between alpha peak amplitude and depressive score in females. Other conventions are as in Fig. 3.


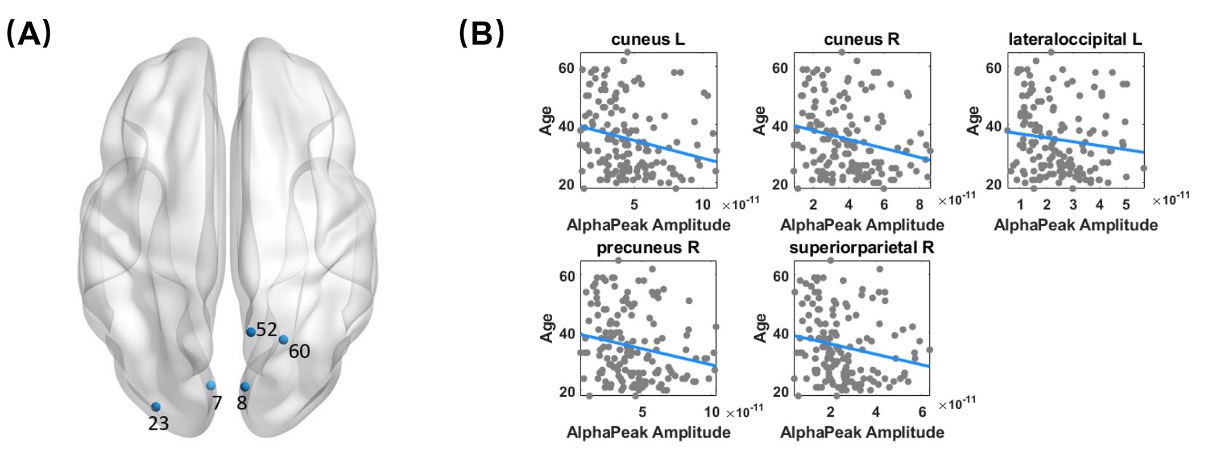


**Fig. S2**. **Illustration of correlation results between alpha peak amplitude and age**. The results revealed a negative correlation between alpha peak amplitude and age. Other conventions are as in Fig. 3.


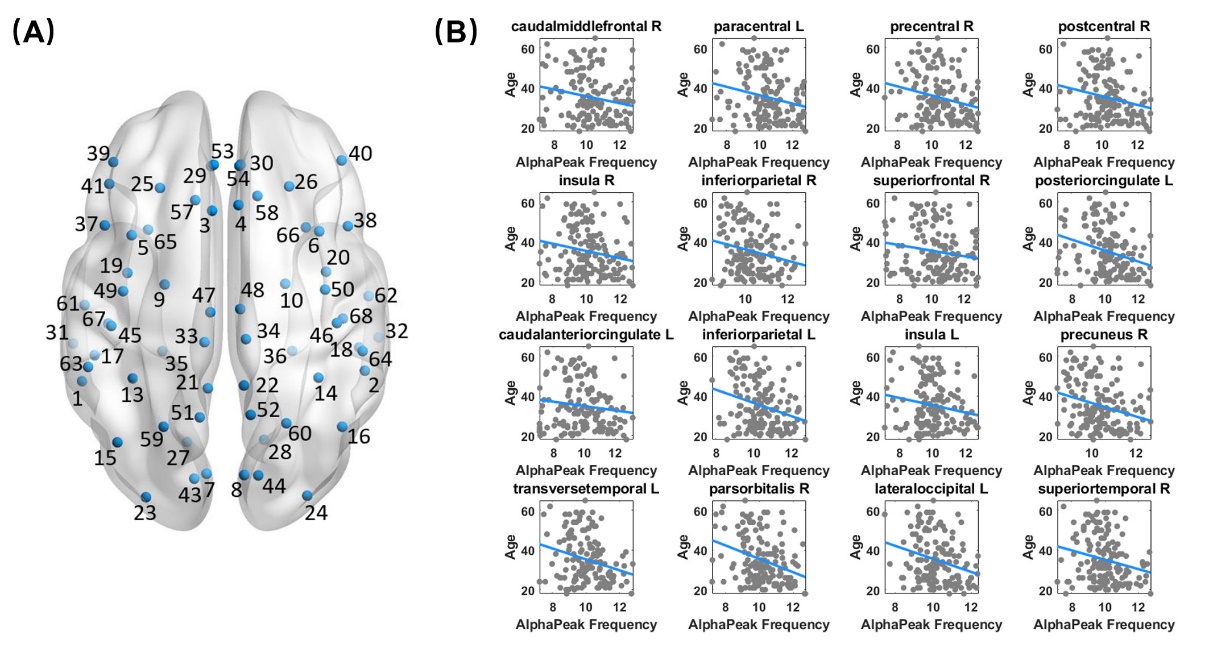


**Fig. S3**. **Illustration of correlation results between alpha peak frequency and age**. The results revealed a negative correlation between alpha peak frequency and age. For clearer data plotting, 16 regions were randomly selected for presentation. Other conventions are as in Fig. 4.

**Table S1.** Brain regions in the Desikan-Killiany atlas. Definitions of the atlas regions were according to Desikan et al. (2006).

| Region Number | Region  Name | Region Number | Region  Name |
| --- | --- | --- | --- |
| 1 | bankssts L | 2 | bankssts R |
| 3 | caudalanteriorcingulate L | 4 | caudalanteriorcingulate R |
| 5 | caudalmiddlefrontal L | 6 | caudalmiddlefrontal R |
| 7 | cuneus L | 8 | cuneus R |
| 9 | entorhinal L | 10 | entorhinal R |
| 11 | frontalpole L | 12 | frontalpole R |
| 13 | fusiform L | 14 | fusiform R |
| 15 | inferiorparietal L | 16 | inferiorparietal R |
| 17 | inferiortemporal L | 18 | inferiortemporal R |
| 19 | insula L | 20 | insula R |
| 21 | isthmuscingulate L | 22 | isthmuscingulate R |
| 23 | lateraloccipital L | 24 | lateraloccipital R |
| 25 | lateralorbitofrontal L | 26 | lateralorbitofrontal R |
| 27 | lingual L | 28 | lingual R |
| 29 | medialorbitofrontal L | 30 | medialorbitofrontal R |
| 31 | middletemporal L | 32 | middletemporal R |
| 33 | paracentral L | 34 | paracentral R |
| 35 | parahippocampal L | 36 | parahippocampal R |
| 37 | parsopercularis L | 38 | parsopercularis R |
| 39 | parsorbitalis L | 40 | parsorbitalis R |
| 41 | parstriangularis L | 42 | parstriangularis R |
| 43 | pericalcarine L | 44 | pericalcarine R |
| 45 | postcentral L | 46 | postcentral R |
| 47 | posteriorcingulate L | 48 | posteriorcingulate R |
| 49 | precentral L | 50 | precentral R |
| 51 | precuneus L | 52 | precuneus R |
| 53 | rostralanteriorcingulate L | 54 | rostralanteriorcingulate R |
| 55 | rostralmiddlefrontal L | 56 | rostralmiddlefrontal R |
| 57 | superiorfrontal L | 58 | superiorfrontal R |
| 59 | superiorparietal L | 60 | superiorparietal R |
| 61 | superiortemporal L | 62 | superiortemporal R |
| 63 | supramarginal L | 64 | supramarginal R |
| 65 | temporalpole L | 66 | temporalpole R |
| 67 | transversetemporal L | 68 | transversetemporal R |

**Table S2.** Significant correlation r values between alpha peak amplitude and depressive score in females.

| Region Name | *r* value |  | Region Name | *r* value |
| --- | --- | --- | --- | --- |
| lateraloccipital R | -0.23 |  | parsorbitalis L | -0.24 |
| lateralorbitofrontal L | -0.28 |  | rostralanteriorcingulate L | -0.21 |
| lateralorbitofrontal R | -0.24 |  | rostralanteriorcingulate R | -0.31 |
| medialorbitofrontal L | -0.29 |  | superiortemporal R | -0.24 |
| middletemporal R | -0.28 |  | temporalpole R | -0.24 |
| parsopercularis R | -0.21 |  |  |  |

**Table S3.** Significant correlation r values between alpha peak amplitude and age.

| Region Name | *r* value |  | Region Name | *r* value |
| --- | --- | --- | --- | --- |
| cuneus L | -0.31 |  | precuneus R | -0.24 |
| cuneus R | -0.29 |  | superiorparietal R | -0.24 |
| lateraloccipital L | -0.23 |  |  |  |

**Table S4.** Significant correlation r values between alpha peak frequency and age.

| Region Name | *r* value | Region Name | *r* value |
| --- | --- | --- | --- |
| bankssts L | -0.23 | parahippocampal L | -0.25 |
| bankssts R | -0.33 | parahippocampal R | -0.32 |
| caudalanteriorcingulate L | -0.18 | parsopercularis L | -0.34 |
| caudalanteriorcingulate R | -0.22 | parsopercularis R | -0.23 |
| caudalmiddlefrontal L | -0.28 | parsorbitalis L | -0.26 |
| caudalmiddlefrontal R | -0.22 | parsorbitalis R | -0.38 |
| cuneus L | -0.25 | parstriangularis L | -0.26 |
| cuneus R | -0.23 | pericalcarine L | -0.27 |
| entorhinal L | -0.24 | pericalcarine R | -0.27 |
| entorhinal R | -0.31 | postcentral L | -0.24 |
| fusiform L | -0.33 | postcentral R | -0.25 |
| fusiform R | -0.24 | posteriorcingulate L | -0.32 |
| inferiorparietal L | -0.33 | posteriorcingulate R | -0.35 |
| inferiorparietal R | -0.26 | precentral L | -0.26 |
| inferiortemporal L | -0.27 | precentral R | -0.20 |
| inferiortemporal R | -0.30 | precuneus L | -0.27 |
| insula L | -0.21 | precuneus R | -0.31 |
| insula R | -0.22 | rostralanteriorcingulate L | -0.23 |
| isthmuscingulate L | -0.30 | rostralanteriorcingulate R | -0.28 |
| isthmuscingulate R | -0.31 | superiorfrontal L | -0.30 |
| lateraloccipital L | -0.25 | superiorfrontal R | -0.19 |
| lateraloccipital R | -0.34 | superiorparietal L | -0.33 |
| lateralorbitofrontal L | -0.28 | superiorparietal R | -0.27 |
| lateralorbitofrontal R | -0.33 | superiortemporal L | -0.32 |
| lingual L | -0.23 | superiortemporal R | -0.31 |
| lingual R | -0.30 | supramarginal L | -0.28 |
| medialorbitofrontal L | -0.23 | supramarginal R | -0.25 |
| medialorbitofrontal R | -0.42 | temporalpole L | -0.33 |
| middletemporal L | -0.29 | temporalpole R | -0.32 |
| middletemporal R | -0.25 | transversetemporal L | -0.33 |
| paracentral L | -0.27 | transversetemporal R | -0.30 |
| paracentral R | -0.21 |  |  |

**Table S5.** Significant correlation r values between alpha peak amplitude and alpha peak frequency. Correlation analysis between alpha peak amplitude and frequency were conducted at each region and the 36 regions showing significant correlations (*p*_corrected_<.05) are listed.

| Region Name | *r* value | Region Name | *r* value |
| --- | --- | --- | --- |
| bankssts L | -0.22 | parsopercularis L | -0.22 |
| bankssts R | -0.16 | parsopercularis R | -0.29 |
| caudalanteriorcingulate L | -0.33 | parsorbitalis L | -0.19 |
| caudalanteriorcingulate R | -0.33 | parsorbitalis R | -0.27 |
| caudalmiddlefrontal R | -0.22 | parstriangularis L | -0.28 |
| entorhinal L | -0.38 | postcentral L | -0.21 |
| entorhinal R | -0.30 | precentral R | -0.17 |
| fusiform L | -0.22 | rostralanteriorcingulate L | -0.36 |
| fusiform R | -0.18 | rostralanteriorcingulate R | -0.29 |
| inferiortemporal L | -0.27 | superiorfrontal L | -0.18 |
| inferiortemporal R | -0.20 | superiorfrontal R | -0.21 |
| insula L | -0.25 | superiortemporal L | -0.30 |
| lateralorbitofrontal L | -0.19 | superiortemporal R | -0.32 |
| lateralorbitofrontal R | -0.30 | supramarginal R | -0.26 |
| medialorbitofrontal L | -0.34 | temporalpole L | -0.37 |
| medialorbitofrontal R | -0.31 | temporalpole R | -0.22 |
| middletemporal L | -0.30 | transversetemporal L | -0.18 |
| middletemporal R | -0.24 | transversetemporal R | -0.27 |

**Supplementary Text**

**Correlation between EEG and clinical measures for males and females**

The analyses were the same as that in the main text, except that the correlation was conducted for males and females, respectively (Tement et al., 2016). For males, no significant correlation was found between the alpha peak amplitude and SDS score or between the alpha peak frequency and SDS score. For females, significant negative correlations were found between the alpha peak amplitude and SDS score at 11 source regions (*p*_corrected_<.05) (Fig. S1) (Table S2). No significant correlation was found between the alpha peak frequency and SDS score.

**Correlation between EEG measures and age**

We further performed correlation analyses between EEG measures and age. Other analysis settings were the same as the analyses in the main text. For alpha peak amplitude, significant negative correlations were found at 5 source regions (*p*_corrected_<.05) (Fig. S2) (Table S3). For alpha peak frequency, significant negative correlations were found at 63 source regions (*p*_corrected_<.05) (Fig. S3) (Table S4).
